# Artificial Light at Night Alters Photosynthetic Electron Transport in Two Deciduous Species

**DOI:** 10.3390/biology15030272

**Published:** 2026-02-03

**Authors:** Monika A. Czaja, Anna Kołton

**Affiliations:** 1 Department of Ornamental Plants and Garden Art, Faculty of Biotechnology and Horticulture, University of Agriculture in Krakow, 31-120 Krakow, Poland; 2 Department of Botany, Physiology and Plant Protection, Faculty of Biotechnology and Horticulture, University of Agriculture in Krakow, 31-120 Krakow, Poland

**Keywords:** light pollution, photo pollution, OJIP, urban trees

## Abstract

Although light pollution is one of the fastest-growing environmental problems today, we still know little about its impact on specific organisms. Plants are the least understood group in this context. Although environmental observations may suggest some conclusions, we still need more specific experimental data. The present study involved two deciduous species—common beech (*Fagus sylvatica* L.) and white dogwood (*Cornus alba* L.)—which, after being placed in growth room, were exposed to additional light at night. The effect of two different nighttime lighting combinations on leaf physiology was analyzed by measuring chlorophyll fluorescence. Nighttime lighting caused disruptions in electron transport, as evidenced by the reduced RE_0_/ET_0_, RE_0_/ABS, and PI_total_ parameters. This result confirms the negative impact of light pollution on plant functioning and provides a basis for further, more in-depth research.

## Simple Summary

Light pollution is one of the fastest growing environmental issues of our time. While ubiquitous light was once thought to improve our functioning as a society, increasing safety and efficiency, it has turned out to be a major ecological problem. Artificial light at night disturbs the natural day–night cycle, and the lack of a dark phase interferes with the functioning of all living organisms. One of the first described observations in plants was visible changes in leaf development. Spring budburst and autumn leaf fall differ in plants located near artificial light sources compared to plants growing further from the light source or under natural night. These phenomena were described based on visual observations. The in-depth reactions and consequences of light pollution in plants are still not known. In the present study, we examined two woody plant species’ electron transport chain reactions after different night light doses. The results confirmed that light pollution, even in low intensity, can disrupt the functioning of the light phase of photosynthesis in plants. Both species revealed a disturbance in energy transformation. We do not know the impact of long exposure to this factor; however, it may lead to serious disturbances in plant functioning.

## 1. Introduction

Light is a crucial factor for plant growth and development. Sunlight is the main source of energy captured during photosynthesis and is used for carbohydrate production, and it is also a signal recognized by plants’ photoreceptors that regulate growth and development [1,2]. The complicated subject of light as a signal includes the quality of light, its direction, intensity, and the changes in light period during day and year [3,4]. The length of the light and dark periods in the daily cycle is called the photoperiod. The reaction of the organism to changes in the length of the light and dark phases is called photoperiodism. Plants can recognize the changes in daylength (light period of the day—24 h), as well as the increase in light intensity during morning and decrease during evening [5,6]. Therefore, light is a universal signal of time and the changing of the seasons [6,7]. Under specific circumstances, it can also be a stress factor for plants: both an excess and deficiency of light represent threats, and a significantly extended photoperiod can also be a stress factor [8]. Naturally, the light signal is only dependent on the Sun’s activity; however, since around 100 years ago, this signal is affected by artificial light emitted from human sources for different reasons. Artificial light at night (ALAN) or light pollution is now recognized as important stress factor for all living organisms on Earth. The problem of light pollution intensifies every year [9,10,11]. In 2023, a Special Issue dedicated to this problem was published in *Science* [12]. Also, in 2020, the Special Issue ‘Sustainable Lighting and Light Pollution’ was published in *Sustainability* [13]. In both examples, the problem of these issues for plants was not a topic that was covered in the collected publications. Regardless of this lack, some studies present observations related to the impact of light pollution on plants [14,15,16].

The first reports on the negative impact of light emitted at night on woody plants were presented in the study of Matzke [17], where delays in leaf fall were observed in plants grown near street lamps in New York. The author presented observations concerning *Populus × canadensis* Moench (Carolina poplar), *Platanus* × *hispanica* Mill. ex Münchh. (London plane), *Acer pseudoplatanus* L. (Sycamore) and *Salix × fragilis* L. (Crack willow). Also, a slower rate of chlorophyll decomposition and unusual leaf breakage points during leaf fall were noted. The impact of light pollution on accelerating spring and delaying autumn phenological phases in plants is well known [14,16,18,19,20]. These processes also depend on day and night temperatures [21], but it has been shown that light pollution has a greater impact on the timing of these phenomena, especially in more urbanized areas [22]. Light at night modifies the architecture of tree crowns [23], the structure of plant leaf blades [24], and the structure and movements of stomata [25]. There is a lack of knowledge about the molecular basis of the phenomena described, as highlighted in the work by Heinen [15].

Photosynthesis is an essential process for the functioning of plants. This process is usually divided into a light-dependent phase and a carbon dioxide fixation phase. Both phases can be disrupted in plants under stressful conditions [26,27]. Various stress factors can affect the functioning of stomata and limit the diffusion of carbon dioxide into the leaf, disrupt the flow of electrons in the light phase, change the chlorophyll content in light-absorbing antennas, or limit the activity of enzymes involved in dark-phase reactions [28]. Under extreme stress conditions, chloroplasts and plant cell membranes may be damaged [29]. Photosynthesis disorders are usually accompanied by the accumulation of reactive oxygen species [29]. Thus, stress factors can affect the process of photosynthesis at various stages.

There are only a few studies on the impact of light pollution on the intensity of gas exchange (photosynthesis) [23,24,30,31] where the photosynthesis was measured both during the day and at night. The measurements were conducted using gas analyzers (carbon dioxide analyzers), which allow conclusions to be drawn about the rate of CO_2_ consumption in the dark phase of photosynthesis and its production in the process of respiration. However, there is a lack of information on the course of light pollution on the light phase of photosynthesis.

The efficiency of the light phase of photosynthesis can be assessed using chlorophyll *a* fluorescence analysis [32]. This method is non-destructive, can be performed on living leaves without damaging them, and allows for the assessment of disturbances in the light phase of photosynthesis when other symptoms are not yet visible [33]. Since 1995, parameters from the JIP test have been increasingly used to assess the impact of stress factors on plants, and it appears that this test can be used for most stress factors [34]. It enables the comparison of the functioning of control tissues and tissues exposed to stress factors. Therefore, this method was chosen to assess the impact of light pollution in our studies.

The aim of the study was to investigate how light pollution treatments influence the electron transport chain in leaves of woody deciduous species, and if different light doses of ALAN would change those reactions. The following research hypotheses were verified:

**Hypothesis** **1.** *Light pollution (LP) causes stress reactions, or at least disorders, in photosynthetic electron transport.*

**Hypothesis** **2.** *Different light doses at night cause different reactions.*

**Hypothesis** **3.** *Species differ in their reaction to LP.*

## 2. Materials and Methods

### 2.1. Plant Material

Two deciduous species, white dogwood (*Cornus alba* L.) and common beech (*Fagus sylvatica* L.), that are popular in European urban ornamental plantings were used in the study. Plants aged 2–3 years old, obtained as rooted cuttings from a local nursery, were potted in the Klassmann TS1 (Klasmann-Deilmann GmbH, Geeste, Germany) commercial substrate, which is recommended for ornamental plants. Potting took place in early-spring 2024, and plants were grown outside until the experiment started. After planting, pots were set for the whole vegetative season at the experimental field of the Faculty of Biotechnology and Horticulture, University of Agriculture in Krakow. The 100 pots with plants of each species were prepared. The plot was localized away from direct light sources at night. Throughout 2024, all plants were treated equally, e.g., watered in summer.

**Figure 1:**
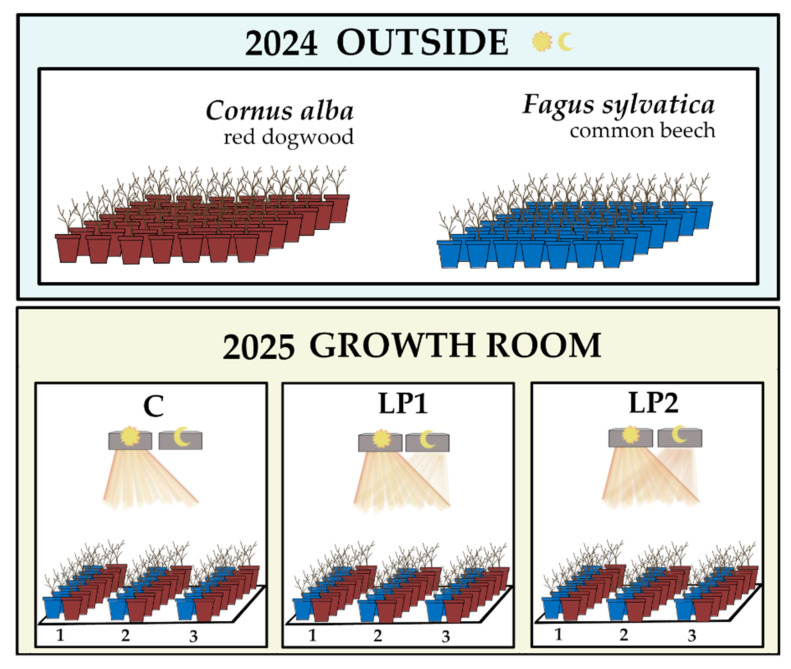
Diagram illustrating the concept of the experiment. In 2024, the plants were placed in outdoor plots, and in 2025, during dormancy, they were transferred to a growth room. There were three isolated spaces with different light conditions (C, LP1, and LP2). The plants were transferred inside three times (1, 2, and 3 are experimental repetitions). The symbols of the Sun and the Moon represent the day and night (or light-polluted) period simulated in the experiment.

The experiment was initiated in early February 2025 (when plants were dormant) and conducted in three repetitions, each commencing two weeks after the previous one. One repetition included 7 plants of each species in every light treatment. The dormant plants were placed in a growth room with a constant temperature (about 20 °C) and humidity (about 60–70%), under three lighting regimes that differentiated between day and night cycles (see Section 2.2) until the development of full leaves. The experimental setup is shown in Figure 1.

### 2.2. Light Pollution Treatment

**Figure 2:**
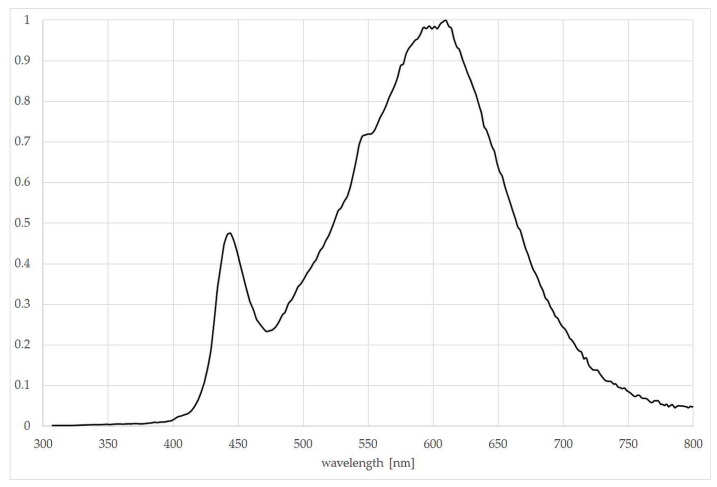
Normalized spectrum from 300 to 800 nm emitted by used LED lamps. The original data was collected as photon flux density with a spectroradiometer (Spectra Pen Mini, Photon Systems Instruments, Drásov, Czech Republic) after dark calibration, in the units µmol·m^−2^·s^−1^·nm^−1^.

The experiment was conducted in a growing room using three separate and unconnected metal frames with shelves. LED lamps were mounted above the shelves on a metal frame, and each entire frame was covered with a light-impermeable material. This arrangement allowed for the control of the light conditions around the plants. The experiment included three light treatments: control plants (C) and two light pollution (LP1 and LP2) combinations. The photoperiod of C was set to a 12 h light period (145 µmol·m^−2^·s^−1^) and 12 h of darkness (0 µmol·m^−2^·s^−1^). For LP1 treatment, the photoperiod was set as 12 h of 144 µmol·m^−2^·s^−1^ and 12 h of 1.5 µmol·m^−2^·s^−1^. For LP2, 12 h of 115 µmol·m^−2^·s^−1^ and 12 h of 30 µmol·m^−2^·s^−1^ was set. Light treatment combinations have different doses of light during the light period to maintain the DLI (daily light integral) value at a similar level, about 6.3 mol·m^−2^·day^−1^ for each treatment (day as 24 h). Light pollution levels can range from 1 µmol·m^−2^·s^−1^ [25] to 700 µmol·m^−2^·s^−1^ [23]. These values vary greatly, which is why we chose two levels that fall within the ranges presented by other researchers, and likely in urban conditions. Light intensity varies significantly depending on the distance from the source. In the present experiment, the LP levels observed directly under (close to) street lamps were not chosen, but both the nighttime light levels that we used are higher than the light of a full moon. The light source was white 4000 K LED lamps set at different heights to obtain the planned intensity at the top of the pot. The spectrum of light emitted by the lamp is presented in Figure 2.

### 2.3. Measurements of Chlorophyll a Fluorescence

Fluorescence measurements were completed after the full development of leaves, which took place approx. after 6 weeks from each replication start for *Cornus* and about 10 weeks for *Fagus.* The chlorophyll *a* fluorescence induction kinetics was measured using a Plant Efficiency Analyzer (Handy-PEA, Hansatech, Norfolk, UK) after 30 min of dark adaptation and a consecutive red-light pulse of 3500 µmol·m^−2^·s^−1^. Measurements were carried out 1 h before the lamps were switched on (−1H), and later, 1 h after turning on the lamps (+1H). Before taking measurements, 10 fully grown leaves were selected and marked. Measurements were taken in 10 replications for each species in every light combination on the same leaves, both prior to and after turning on the lamps.

The collected data were used to present OJIP transient curves and to calculate specific events of the OJIP transients in the OK, OJ, JI and IP phases. The data were double-normalized and presented as the kinetics of relative variable fluorescence and as a different kinetic profile [35,36]. Specific parameters related to quantum efficiencies and flux ratios, as well as performance indexes, were measured and calculated according to Strasser et al. [37].

### 2.4. Data Processing and Statistical Analysis

The total number of measurements per each treatment was 30 (10 leaves × 3 repetitions of the experiment); however, some data were erroneous. Therefore, the raw data was pre-processed and analyzed, and measurements that were found to be faulty were removed. Therefore, an unequal number of results was taken into account when calculating the means (number of data points is 27–30 for dogwood and 18–30 for beech). The data are presented as mean values from three repetitions of the experiment for every specimen in each light treatment (C, LP1, LP2). Statistical comparisons between light treatments were performed separately for −1H and +1H for each species.

Original data were tested for normal distribution with the Shapiro–Wilk test and equality of variances with Levene’s test. After analysis of variance, homogenous groups were separated by Tukey’s test for unequal *n*. Results were considered significant with *α =* 0.05. Statistical analyses were performed using Statistica software, version 13 (TIBCO Software Inc., Palo Alto, CA, USA, software.dell.com).

GenAI was not used in the preparation of this paper.

## 3. Results and Discussion

### 3.1. The OJIP Chlorophyll a Fluorescence Transient Curve and Specific Events in the OJIP Curves

The fast chlorophyll *a* fluorescence kinetics, described as OJIP transient (or OJIP curves), is widely used in detecting different kinds of plant stress [38]. It has been documented as a useful tool in measuring PSII functionality under different environmental stresses, such as drought [39], salt [40], chilling [41], freezing [42], heat [43] or excess heavy metals [44,45]. The in-depth analysis of OJIP curves allows us to conclude the electron flow from the moment of absorption by the PSII antennas to the reduction of PSI [34]. Due to the novel approach of our study concerning the impact of light pollution on the electron transport chain and the scarce literature data in this field, we mainly refer to studies concerning different kinds of environmental stress.

**Figure 3:**
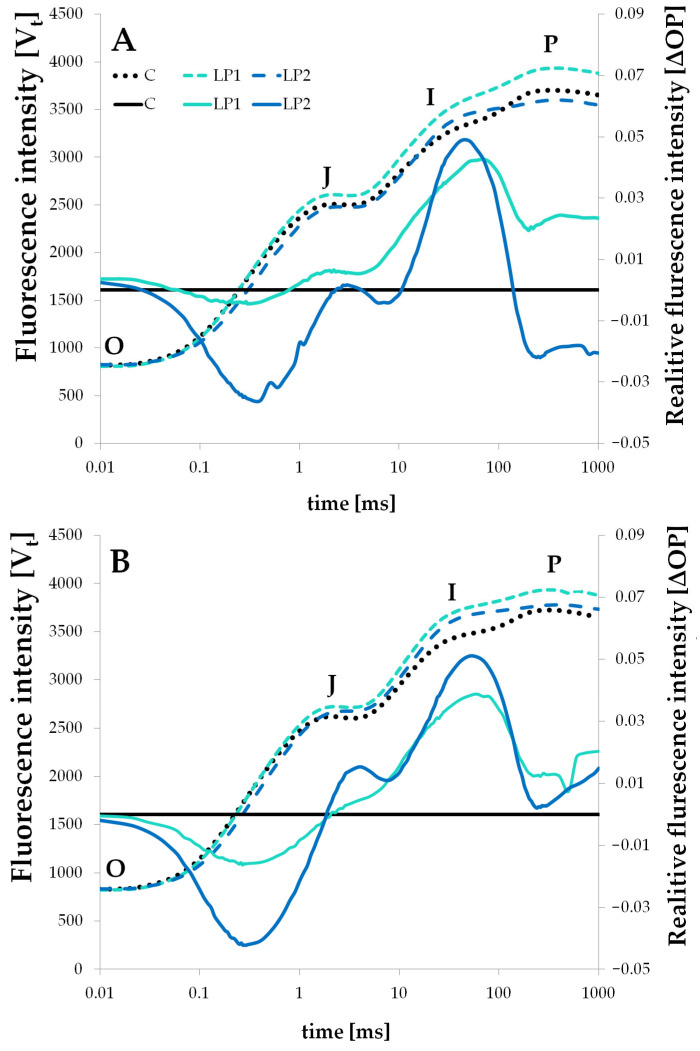
The *F. sylvatica* OJIP chlorophyll *a* fluorescence transient curve (fluorescence intensity V_t_) (dashed lines) and difference kinetics (solid lines) measured 1 h before (**A**) and 1 h after (**B**) turning on the lamps for different light treatments (C—control, LP1—light pollution 1, LP2—light pollution 2). Difference kinetics is presented as relative fluorescence intensity ΔOP. O is fluorescence at 0.02 ms, J is fluorescence at 2 ms, I is fluorescence at 30 ms and P is maximum fluorescence.

**Figure 4:**
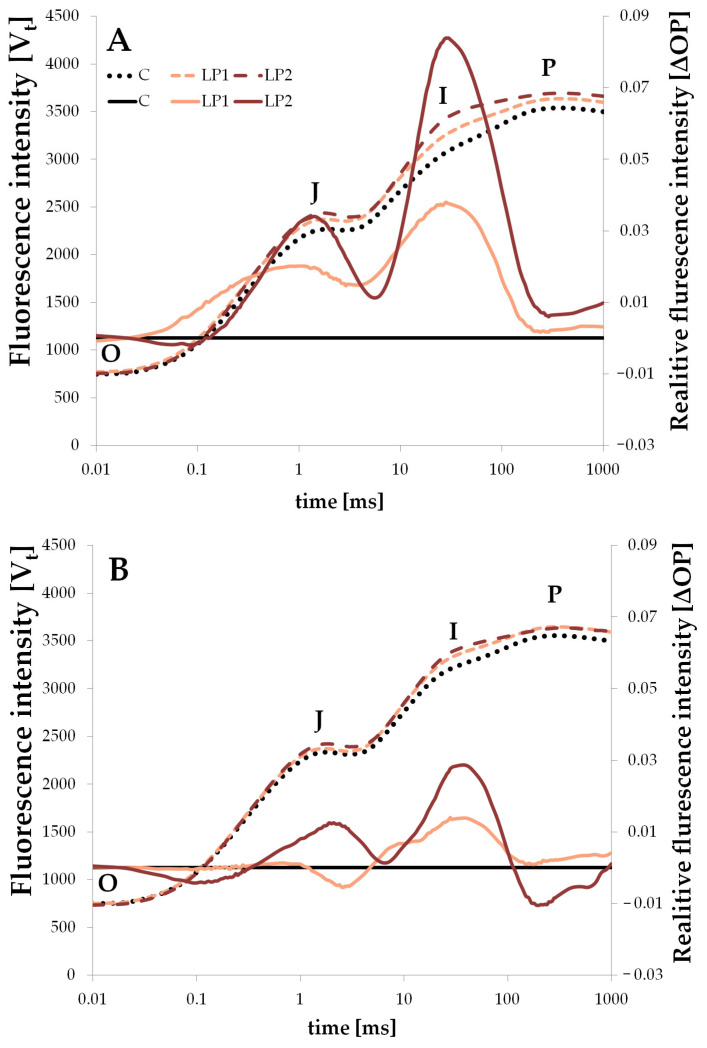
The *C. alba* OJIP chlorophyll *a* fluorescence transient curve (fluorescence intensity V_t_) (dashed lines) and difference kinetics (solid lines) measured 1 h before (**A**) and 1 h after (**B**) turning on the lamps for different light treatments (C—control, LP1—light pollution 1, LP2—light pollution 2). Difference kinetics is presented as relative fluorescence intensity ΔOP. O is fluorescence at 0.02 ms, J is fluorescence at 2 ms, I is fluorescence at 30 ms and P is maximum fluorescence.

In the present experiment, LP treatments resulted in higher OJIP curves than the control ones for both species. Initial fluorescence intensity was similar for all treatments; however, they differentiated in the further measurements (Figure 3 and Figure 4).

Such a rise can be linked to stressful conditions, and indicates a disturbance in normal functioning. A rise in the OJIP curve in the J-step was also observed in peanut plants subjected to drought stress [46], in small-leaved lime under drought and under salinity stress [47], and also in yellow poplar (*Liriodendron tulipifera* L.) under three different night lighting regimes [48]. The authors connected such reactions with disrupted electron transfer from Q_A_ (plastoquinone A) to Q_B_ (plastoquinone B). In our study, only beech measured 1 h before lamps on LP2 had a similar OJIP curve to the control, and that was even lower around the P point (Figure 3A). Other cases present the first symptoms of a disruption in the electron transport chain. Implementing light at night also resulted in positive ΔOP amplitudes after the J points for both LP treatments in the two investigated species (Figure 3 and Figure 4). The rise in the ΔV_t_ compared to control plants was also observed in two woody species under heavy metal stress [49]. Manganese treatment in two doses resulted in a high rise in the ΔV_t_ for deciduous *Melia azedarach* L. and evergreen *Ligustrum lucidum* WT Aiton. Also, in the mentioned study on small-leaved lime (*Tilia cordata* L.), drought stress and salinity stress resulted in the rise in the J-step and I-step of ΔV_t_ [47]. The rise in the relative fluorescence intensity at the J-step relates to the inhibition of the electron transport chain in PSII and the accumulation of Q_A_^−^ [50]. The further rise in the I-step suggests the accumulation of the Q_A_^−^ and Q_B_^−^ [49]. Similar observations were also made under light stress treatments for both investigated species; however, the J-step rise was not as evident in the measurements 1 h before the lamps were switched on in beech (Figure 3A). In beech, relative fluorescence intensity was positive after the J point for LP1 in both measurements (before and after the lamps were turned on), but for LP2, the transient had greater amplitudes—it was more negative than LP1 before the J-step and more positive after the J-step with the highest peak. It is interesting that for beech measured −1H, the ΔOP transient in LP2 (Figure 3A) became negative again around the P point, which was not seen in the measurement for +1H (Figure 3B). Such a decrease was also noted for *Cornus* LP2 measured +1H (Figure 4B). In general, similar observations were made for the *C. alba*, but in this species, the peaks of ΔOP amplitude for both LP treatments measured −1H were visibly higher than those in +1H (Figure 4A,B). The negative amplitude before the J-step is much slighter in *C. alba* than in *F. sylvatica*, and is only visible for the LP2 treatment.

**Figure 5:**
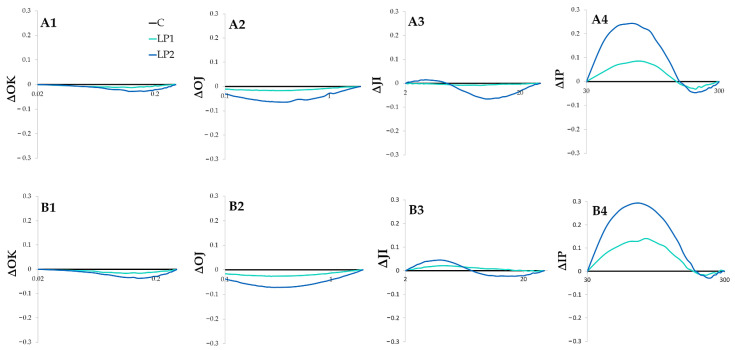
The *F. sylvatica* difference kinetics of the individual bands, ΔOK—L-band (1), ΔOJ—K-band (2), ΔJI—H-band (3) and ΔIP—G-band (4), measured 1 h before turning on the lamps (**A**) and 1 h after (**B**) for different light treatments (C—control, LP1—light pollution 1, LP2—light pollution 2).

**Figure 6:**
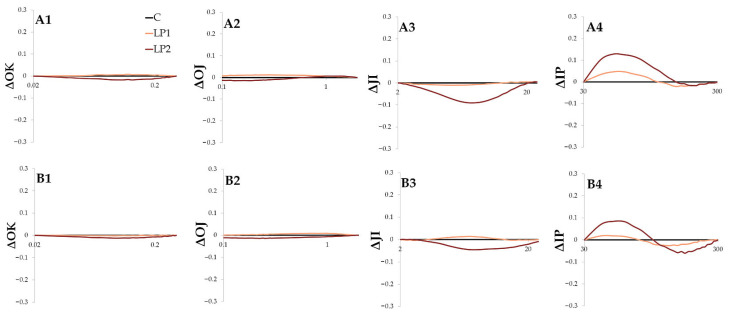
The *C. alba* difference kinetics of the individual bands: ΔOK—L-band (1), ΔOJ—K-band (2), ΔJI—H-band (3) and ΔIP—G-band (4) measured 1 h before turning on the lamps (**A**) and 1 h after (**B**) for different light treatments (C—control, LP1—light pollution 1, LP2—light pollution 2).

There were no visible L, K or H bands (ΔOK, ΔOJ and ΔJI, respectively) for the investigated species in both LP treatments (Figure 5 and Figure 6). The lack of those bands may suggest that the implemented light pollution treatments did not disrupt photosystem stability, the oxygen-evolving complex, or the oxidation of plastoquinone [51]. However, both investigated species reveal positive G bands (ΔIP) in light pollution treatments (Figure 5(A4,B4) and Figure 6(A4,B4)), suggesting photosystem disturbance in the final stages of electron transport. The decrease in the IP phase, presented as ΔIP, is an effective indicator of plant performance and reflects the content of PSI reaction centers [52]. High values for the IP phase were linked with the photo-oxidative protection mechanisms observed in trees under high irradiance levels [53]. In the study, concerning light-demanding *Betula pendula* Roth and *Pinus sylvestris* L. and the shade-tolerant tree species *Picea abies* L., the increase in the IP phase was connected to high solar radiation and heat stress [54]. Also, the prolonged photoperiod in a short-day *cannabis* plant resulted in a positive G-band [55], which the authors interpreted as a decrease in the PSI pool. The positive G band can also be interpreted as reduced electron transport and a consequent decrease in the rate of PSI reduction [51]. The light pollution implemented in the present study caused a similar reaction to beech leaves under high radiation stress [56]. The IP-phase rise can be related to the disruption of the electron flow and energetic pressure in the PSI side, as it reflects the reduction of the PSI electron acceptor [50]. The PSII is thought to be primarily affected by different unfavorable factors leading to reduction in the photosynthetic efficiency. However, there is some evidence for more complex differences among species and under various environmental conditions [52]. For example, two deciduous trees, *Acer saccharinum* L. and *Quercus bicolor* Willd., revealed a preferential reduction of PSI rather than PSII during autumnal senescence [57]. While chlorophyll *a* fluorescence was considered to mostly screen for PSII functioning, there is more evidence that PSI disruption is also visible in the OJIP curves, mostly in the IP phase [58]. Under light stress and the associated photo-inhibition, PSI becomes inactivated with reported long regeneration time [59]. In the present study, neither ROS accumulation nor P700 oxidation state were analyzed. Therefore, the damage of PSI due to photo-inhibition was not confirmed; however, this could be considered in future long-term studies. It needs to be underlined that in cases of light pollution, the stress event is not severe and occasional (as it is in the case of high radiation stress or prolonged photoperiod studies), but rather moderate and permanent; therefore, the regeneration of PSI would be difficult. The IP bands had higher positive amplitudes for *F. sylvatica* than for *C. alba*, and in *C. alba* +1H, the band tends to be partially negative (Figure 6(B4)). This may underline the differences between species and highlights the need for more complex studies involving more tree species to distinguish between light-pollution-tolerant and -sensitive ones.

### 3.2. JIP Test Parameters

**Figure 7:**
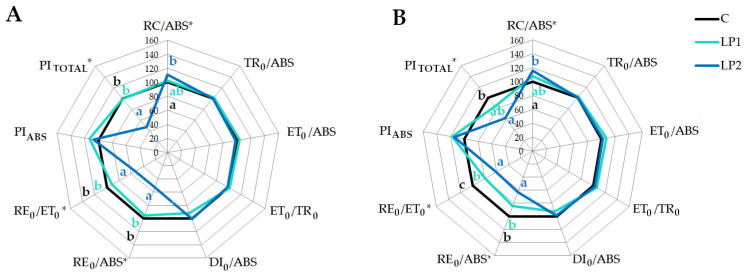
The JIP test parameters derived from OJIP curves, presented for *F. sylvatica* in two terms of measurements—1 h before lamps were turned on (**A**) and one hour after (**B**). Results for control plants were set as 1, and both LP treatments are presented in relation to the control. Statistical differences if detected were marked as *, and different letters mean statistical groups according to Tukey’s test with *p* < 0.05.

**Figure 8:**
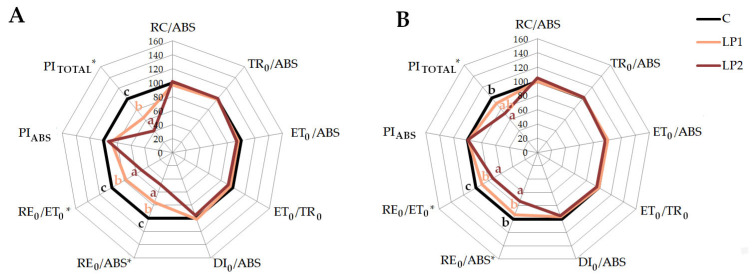
The JIP test parameters derived from OJIP curves, presented for *C. alba* for two terms of measurements—1 h before lamps were turned on (**A**) and one hour after (**B**). Results for control plants were set as 1, and both LP treatments are presented in relation to the control. Statistical differences, if detected, were marked as *, and different letters mean statistical groups according to Tukey’s test with *p* < 0.05.

The individual parameters calculated from the JIP test correspond with the described OJIP curves structure. The main parameters describing the PSII efficiency and vitality such as PI_ABS_ or TR_0_/ABS did not show visible changes under light pollution treatments compared to control (Figure 7 and Figure 8) for both investigated species. The disruption at the beginning of the electron transport chain is reported in plants subjected to stress [36]. However, we did not record changes at this point of electron transport. Fluorescence parameters such as TR_0_/ABS and ET_0_/ABS reflect the probability that the absorbed photons lead to a reduction in Q_A_ and the probability and efficiency of electron transport further than Q_A_, respectively [60]. In our study, we did not observe the disturbance of those parameters. There was also no increase in the DI_0_/ABS parameter, which is connected to the dissipation of energy in PSII in the literature [61].

The significant growth of active reaction centers (RC/ABS) under LP2 compared to control was only found in beech (Figure 7). Such a reaction may be connected to a higher amount of active reaction centers [32,62], but also reduction in specific energy fluxes such as absorbance [63]. The reduction in the antenna size and the greater number of active reaction centers under energy disturbance delays the photo-oxidative damage. It is interesting that such reactions are observed under high light stress to PSII damage by regulating the RC density [64]. However, both investigated species show disturbances in the parameters connected with the reduction of the PSI acceptor (RE_0_/ABS) and electron transport to the PSI end-acceptor (RE_0_/ET_0_). Values of the RE_0_/ET_0_ are related to the electron transferred from the PQH_2_ (plastoquinol) to the PSI and the electron influx from the upper carrier [65]. A disturbance in the PSI electron transport, presented as a decrease in RE_0_/ET_0_ or RE_0_/ABS, was also observed under different environmental stresses in trees. *P. sylvestris* subjected to drought stress [66] revealed a decrease in RE_0_/ET_0_, *Malus domestica* Borkh. ‘Honeycrisp’ with zonal chlorosis [67] showed a RE_0_/ABS decrease, and both parameters decreased in *Citrus grandis* treated with different Al solutions [68]. However, under stressful conditions which disrupt PSII, plants often activate cyclic electron flow to reduce end-acceptors of PSI. As mentioned by the authors, such a reaction prevents the severe damage of PSI due to the production of ROS (reactive oxygen species), which results in an increase in the RE_0_/ET_0_, as observed in the *Lonicera japonica* Thunb. under heat stress [69] and in *Malus hupehensis* Rehder infected by *Fusarium* [70]. It is concerning that under light pollution, we observed a decrease in the RE_0_/ET_0_ in both investigated species, regardless of the measurement time. This may suggest that light pollution conditions are not severe enough to activate the photo-protective mechanism, but on the other hand, according to the cited references, they may lead to severe disruption in the PSI. Therefore, long-term consequences of such stress need to be investigated. As shown for yellow poplar, the maximum quantum yield of PSII chemistry was only significantly decreased after 28 months of light pollution treatment [48].

Previous studies proved that prolonged excessive light stress strongly affects PI_ABS_ values [71]. However, it needs to be underlined that the reaction to light pollution would not be the same as this in cases of high light stress. In the present study, the PI_ABS_ did not change significantly for any species (Figure 7 and Figure 8). Energy conservation from absorbed photons to the reduction of intersystem carriers was not affected. However, we noted a significant decrease in PI_TOTAL_ in both species for LP2 compared to the control. PI_TOTAL_ reflects the efficiency of energy conservation in the reduction of PSI acceptors, which confirms our previous assumptions about PSI disruption.

### 3.3. Diurnal Light Conditions

Plants exhibit metabolic, physiological, and developmental processes that vary throughout the day, depending on the diverse expression of genes and protein activity [72]. It has been shown that sensitivity and response to stress factors vary depending on the time of day [73,74]. The response of plants to temperature stress occurring immediately after the start of the photoperiod activates a different set of genes than when this stress occurs in the afternoon [75]. Alternatively, the level of expression may be different if the same genes are activated. The time of day was also important in the responses of *Arabidopsis* plants to drought stress [76]. Similarly, it has been shown that the time of day affects the response of *Populus* to drought stress [77], and the authors explicitly state that it is impossible to determine the full picture of plant responses to a given factor after collecting samples at a single time point. Kwak et al. [48] measured the OJIP curve throughout the light period of a day, showing that the greatest deviation of samples treated with light at night from the control is in the morning, while in the evening, the curves are similar to the control. In our study, we recorded the curves one hour before the end of the photoperiod (end of night) and one hour after the start of the photoperiod (morning). For the dogwood, the differences between LP and control plants were more evident at the end of the night period (Figure 5 and Figure 6). In the future, the diurnal pattern of plant response to light pollution should be determined.

Hypothesis 1 was confirmed with the disturbances of electron transport under light pollution treatment. However, despite our expectations, there was no detected disorder in the PSII signal transduction, which was observed in other studies concerning environmental stresses. The recorded disturbances of PSI functioning were similar for both investigated species, and confirmed stressful conditions under artificial light pollution.

Hypothesis 2 was confirmed in both OJIP curves and specific parameters; however, the course of changes was similar, and the intensity of the light at night was also an important factor. In most cases, a higher intensity of light implemented at night caused the most severe reactions.

Hypothesis 3 was partially confirmed; while species differed in the specific OJIP curves course, the main tendency was similar. Further analysis is necessary to distinguish between species that are more susceptible and resistant to light pollution.

## 4. Conclusions

We confirmed that artificial light at night cause disruption in photosynthetic electron transport, even in small light doses such as 1.5 µmol·m^−2^·s^−1^. In general, more intensive light doses (30 µmol·m^−2^·s^−1^) during the night phase triggered more severe reactions. The disturbance in electron transport observed in PSI can lead to physiological dysfunction at other levels of plant functioning. More detailed studies concerning further reactions need to be undertaken. It needs to be underlined that the plants in this study were subjected to light pollution treatment for a relatively short time period (about 6 to 10 weeks), and that the leaves were tested at the onset of their full development. Furthermore, we presented slight differences between investigated species’ reactions, but more in-depth studies including more taxa are needed to distinguish between light-pollution-susceptible and -tolerant species.

## Data Availability

The data presented in this study are available on request from the corresponding author.
